# Educational level and 30-day outcomes after hospitalization for acute myocardial infarction in Italy

**DOI:** 10.1186/s12913-016-1966-5

**Published:** 2017-01-09

**Authors:** Gianluca Cafagna, Chiara Seghieri

**Affiliations:** Health and Management Laboratory (MeS Lab), Institute of Management, Sant’Anna School of Advanced Studies, Piazza Martiri della Libertà, 24, Pisa, Italy

**Keywords:** Education, Socioeconomic status, Acute myocardial infarction, Short-term mortality, Short-term readmission, Italy, Health care outcomes, Hospital performance, Health equity, Health services research

## Abstract

**Background:**

There is a growing interest in the factors that influence short-term mortality and readmission after hospitalization for acute myocardial infarction (AMI) since such outcomes are commonly considered as hospital performance measures. Socioeconomic status (SES) is one of the factors contributing to healthcare outcomes after hospitalization for AMI. However, no study has been published on education and 30-day readmission in Europe. The objective of this study is to examine the association between educational level and 30-day mortality and readmission among patients hospitalized for AMI in Tuscany (Italy).

**Methods:**

A retrospective cohort study using data from hospital discharge records was conducted. The analysis included all patients discharged with a principal diagnosis of AMI between January 1, 2011, and November 30, 2014, from all hospitals in Tuscany. Educational level was categorized as low (no middle school diploma), mid (middle school diploma) and high (high school diploma or more). Three multilevel models were developed, sequentially controlling for patient-level socio-demographic and clinical variables and hospital-level variables. Patients were stratified by age (≤75 and >75 years).

**Results:**

Mortality analysis included 23,402 patients, readmission analysis included 22,181 patients. In both unadjusted and full-adjusted models, patients with a high education had lower odds of 30-day mortality compared to those patients with low education (OR age ≤ 75 years 0.67, 95% CI:0.47–0.94; OR age > 75 years 0.72, 95% CI:0.54–0.95). With regard to 30-day readmission, only patients aged over 75 years with a high education had lower odds of short-term readmission compared to those patients with low education (OR age > 75 0.73, 95% CI:0.58–0.93).

**Conclusions:**

Among patients hospitalized in Tuscany for AMI, low levels of education were associated with increased odds of 30-day mortality for both age groups and increased odds of 30-day readmission only for patients aged over 75 years. Our findings suggest that the educational component should not be underestimated in order to improve short-term outcomes, which are considered as performance measures at the hospital level. Hospital managers might consider strategies that are sensitive to patients with low SES, such as providing post-hospitalization support to less-educated patients and promoting a healthier lifestyle, to improve both health equity and performance outcomes.

## Background

Since 30-day outcomes after hospitalization for acute myocardial infarction (AMI) are commonly considered as hospital performance measures [1–3], hospitals and clinicians have shown increasing interest in understanding and improving factors associated with 30-day mortality and readmission rates [4, 5]. Evidence suggests that short-term mortality for AMI, heart failure, and pneumonia closely predict short-term mortality for a variety of other surgical and medical conditions, thus highlighting the utility of these measures to reflect a broader quality of hospital performance [6].

Short-term readmission is also a proxy of avoidable adverse health outcomes with relevant cost implications. A literature review highlights that between 5 and 59% of short-term readmission could be avoided [7], while the cost to Medicare of unplanned readmissions was estimated to be $17.4 billion per year [8]. Hence, efforts to deepen the understanding of factors that influence early mortality and readmission for AMI patients could help to both improve quality and reduce costs.

Socioeconomic status (SES) is one of the factors contributing to poor health care outcomes after hospitalization for AMI [9–13]. The educational level has been used as a proxy for SES and is a strong predictor of both short-term [4, 14–20] and long-term outcomes [21–23]. Several studies have analysed the association between education and short-term mortality, but evidence is still needed in universal health care systems, which should guarantee equity in health by mandate. Furthermore, to the best of our knowledge no evidence has been provided on education and short-term readmission in Europe.

In 2005, Tuscany became one of the few regions in Italy to develop a Performance Evaluation System (PES) based on the systematic collection of hospital performance indicators from administrative data sources [24]. Equity indicators were included in the Tuscan data monitoring process in 2008, and educational status was part of the SES information monitored in the hospitalization records of all Tuscan providers. In 2010 equity indicators were introduced into the Tuscan PES and in the planning and strategic control system of the Tuscan health care organizations [25]. The Tuscan PES is increasingly developing its equity dimension, making Tuscany an optimal location for carrying out the present study.

This research aims to provide context-specific evidence to extend the knowledge on and improve the understanding of SES factors associated with short-term outcomes after hospitalization for AMI. We therefore examined the association between educational level and 30-day mortality and readmission among patients hospitalized for AMI in Tuscany.

## Methods

### Data

We conducted a retrospective cohort study using data from hospital discharge records (HDRs). The analysis included all patients discharged with primary International Classification of Diseases, 9th revision, Clinical Modification (ICD-9-CM) codes of AMI between January 1, 2011, and November 30, 2014, from all hospitals in Tuscany. HDR data quality is routinely checked by the Regional Health Information System Office, which provides hospitals with feedback on missing data and logical inconsistencies.

Records were excluded based on criteria previously used elsewhere [26, 27]:

1. Admissions lasting less than two days – due to concerns about the accuracy of the diagnosis;
2. Admissions of patients not resident in Tuscany;
3. A diagnostic code 410.9 – AMI of unspecified site;
4. A diagnostic code 410.x2 – subsequent “episode of care” for discharged patients
5. Admissions of patients under 18 years and over 100 years;
6. Admissions of patients discharged from hospitals with less than 10 AMI admissions per year.

**Fig. 1:**
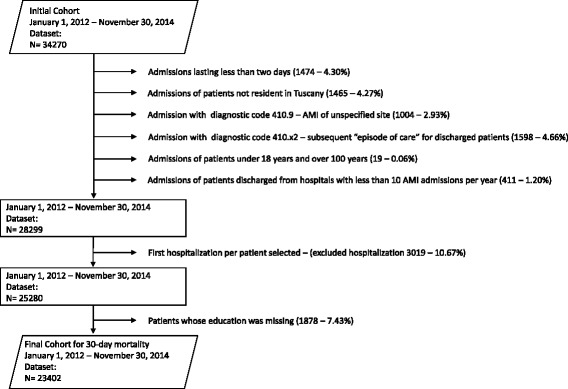
AMI Cohort exclusions in the January 2012-November 2014 Dataset

In order to avoid selection bias, we selected the first AMI episode [14, 15, 17], and only included patients that had a baseline evaluation of 1 year with no AMI. For the readmission analysis, we considered only unplanned readmission and excluded patients only if they died during hospitalization (1221), not if they were alive at least 30 days after discharge, in line with similar studies, the Centers for Medicare & Medicaid Services, and the Tuscan Outcome Program [9, 28, 29]. In cases where patients incurred more than one admission during the first 30 days after discharge, we considered only the first readmission. If patients were transferred, both mortality and readmission were attributed to the hospital to which the patients were initially admitted [27]. In accordance with similar studies [14, 20], we excluded 1878 patients whose educational level had not been registered. The inclusion/exclusion for the mortality analysis are shown in Fig. 1.

The Regional Health Information System Office assigned each patient with a unique identifier, which was the same for all administrative databases, and does not disclose the patient’s identity or other sensitive data. The study was performed in full compliance with Italian privacy laws. Approval by an Ethics Committee was unnecessary, given that we used data from HDRs.

### Outcomes and exposures

In a departure from longitudinal studies, our outcomes of interest were short-term outcomes after AMI, which are more relevant from a hospital performance perspective. 30-day mortality was defined as a death occurring due to any cause either during or after hospitalization within 30 days from the index admission; 30-day readmission was defined as a rehospitalisation occurring for any cause within 30 days of discharge. Information on mortality was obtained through a deterministic record linkage procedure between HDRs and the Tuscan residence registry, using the unique patient identifier. Similarly, follow-up was via record linkage of HDRs from all hospitals in Tuscany, using the unique patient identifier.

Data elements included socio-demographic variables, clinical variables and hospital variables. Socio-demographic variables (age, sex, and SES) were collected from the regional HDRs. We used the patient’s individual level of education as a proxy for individual SES. Educational level was categorized as follows: low education (no middle school diploma), mid education (middle school diploma) and high education (high school diploma or more). These categories were defined on the basis of the birth cohort [30]. Given that patients’ median age was 74 years and just 3.12% of patients included in the mortality analysis had a university degree, it seemed reasonable to consider both people with a high school diploma and people with a university degree under the same category (high education).

Clinical variables included the presence or absence of previous AMI episodes, type of ST-segment elevation (STEMI/NSTEMI), and specific patient comorbidities. The type of ST-segment elevation was the best proxy available to us for AMI severity. The ICD-9-CM codes recorded in the previous 2 years were used to define specific patient comorbidities (tumours, diabetes, hematologic diseases, hypertensive diseases, other forms of ischemic heart diseases, heart failures, other cardiac conditions, conduction disorders and cardiac dysrhythmias, cerebrovascular diseases, vascular diseases, chronic obstructive pulmonary disease, and chronic nephropathies).

Hospital variables (teaching status and presence or absence of cardiac catheterisation laboratory) were included in the analysis, because they might confound the association between education and short-term outcomes. A cardiac catheterisation laboratory operates in hospitals equipped with relevant surgical support, and provides diagnosis and therapy services for various cardiovascular diseases [27].

### Statistical analysis

Hierarchical logistic regression models, also known as multilevel models, were fitted due to the hierarchical structure of our data (i.e. patients nested within hospitals), in line with other studies [9]. In fact, “multilevel modes are used to take into account the effects of clustering of patients into hospitals and of hospital characteristics, that may affect short-term mortality” [27]. 30-day mortality and 30-day readmission are short-term outcomes considered as performance measures at the hospital level. We were interested in these outcomes because our final aim is to contribute to making hospital managers aware of the influence of individual SES on performance measures, which are used in public reporting. On a similar note, we decided to perform logistic regression models and not survival models because public reporting authorities, such as the Centers for Medicare & Medicaid Services in the USA [29], and National Outcome Program in Italy [31, 32], perform logistic models to estimate risk-adjusted short-term mortality or readmission rates. Moreover, 30-day mortality and 30-day readmission are dichotomous outcomes, indicating whether or not the patient died or was readmitted within 30 days. Logistic regression models match the dichotomous nature of our outcomes and have been extensively used by other scholars for short-term outcomes [9, 15, 31]. In order to test an alternative specification, we also performed survival models. The results using survival models were consistent with the results using logistic models.

Three two-level models were fitted for both the mortality and readmission analysis. The first model was unadjusted, including only the patients’ individual level of education; the second model was adjusted for patients’ age, sex, and clinical characteristics; the final model also included hospital characteristics. The analysis was stratified into two age groups: under 75 years and over 75 years. Hierarchical logistic regression models yielded odds ratios (ORs) with 95% confidence intervals (CIs) for both patient-level and hospital-level variables. In order to estimate ORs for the individual level of education, we used low education as the reference category.

We also performed a specific analysis to assess the number of patients hospitalized for AMI in Tuscany and readmitted within 30 days of discharge in other Italian regions. It turned out that, over the study period, just 50 patients (0.22%) were readmitted elsewhere in Italy, having no impact on our results. The limited number of Tuscan patients readmitted in other Italian regions could be explained by the fact that readmissions might be related to unplanned health events or that the Tuscan health care system is recognized as one of the best in Italy [33] and thus patients prefer to receive health care services in Tuscany.

*P* value <0.05 was considered significant, and confidence intervals were calculated at 95%. All analyses were carried out using SAS for Windows, version 9.3 (SAS Institute, Cary, NC) and STATA, version 13 (StataCorp LP, College Station, TX).

## Results

### Baseline

The mortality analysis included 23,402 patients; the readmission analysis included 22,181 patients. HDRs were extracted from 34 hospitals, of which 16 (47.1%) had a cardiac catheterization laboratory and 5 (14.7%) were teaching hospitals. The overall crude 30-day mortality was 7.0%, 2.5% among patients under 75 years, and 12.1% among those over 75 years. The overall crude 30-day readmission was 12.3%, 9.6% among patients under 75 years, and 15.7% among those over 75 years.

Patients with a low education had a significantly higher 30-day mortality and readmission than patients with a high education, in both age groups. In addition, patients with a lower education were significantly older, more often women, had more comorbidities, had more previous AMI episodes, and had more STEMI type of AMI compared to patients with higher education.

Tables 1 and 2 show the patients’ characteristics (clinical and demographic) stratified by age and educational level for mortality and readmission analysis, respectively.

**Table 1:** Mortality analysis: baseline characteristics of study population, stratified by age and education

	Overall					age ≤75					age > 75				
	Level of education					Level of education					Level of education				
	All	Low	Mid	High	*p*	All	Low	Mid	High	*p*	All	Low	Mid	High	*p*
Number	23,402	12,613	6667	4122		12,409	4128	4924	3357		10,993	8485	1743	765	
%	100	53.9	28.5	17.6		100	33.3	39.7	27.0		100	77.2	15.9	7.0	
Demographic variables															
Age in years	71.7	78.0	65.6	62.4	*	61.6	66.6	59.9	57.9	*	83.2	83.6	81.7	81.9	*
Male (%)	63.6	53.4	73.2	79.4	*	76.1	68.3	78.5	82.2	*	49.9	46.1	57.9	66.8	*
Clinical variables															
Tumours (%)	5.6	6.1	5.4	4.7	*	4.5	5.3	4.4	3.7	*	6.9	6.4	8.1	9.0	*
Diabetes (%)	8.8	10.9	7.5	4.4	*	6.2	9.0	5.8	3.3	*	11.7	11.8	12.2	9.4	
Hematologic diseases (%)	6.7	8.9	4.6	3.2	*	2.9	4.0	2.7	2.1	*	10.9	11.3	10.2	8.0	*
Hypertensive diseases (%)	14.3	17.3	12.0	8.9	*	9.4	11.7	9.1	6.9	*	19.9	20.0	20.4	17.5	
Other forms of ischemic heart diseases (%)	11.6	13.7	10.0	7.7	*	8.0	10.0	7.7	6.2	*	15.7	15.6	16.8	14.2	
Previous heart failures (%)	6.8	9.1	4.6	3.0	*	3.0	4.4	2.5	1.9	*	11.1	11.5	10.5	7.7	*
Other cardiac conditions (%)	2.6	3.2	2.0	1.9	*	1.4	2.0	1.2	0.9	*	4.1	3.8	4.2	6.3	*
Conduction disorders and cardiac dysrhythmias (%)	6.7	8.9	4.7	3.2	*	2.9	4.3	2.5	1.7	*	11.0	11.1	11.2	9.5	
Cerebrovascular diseases (%)	9.3	11.8	7.2	4.9	*	4.7	6.3	4.5	3.2	*	14.4	14.5	14.9	12.7	
Vascular diseases (%)	7.3	8.5	6.5	4.8	*	5.8	7.6	5.5	4.0	*	9.0	8.9	9.5	8.4	
Chronic obstructive pulmonary disease (%)	4.2	5.4	3.4	1.8	*	2.3	3.4	2.2	1.2	*	6.3	6.4	6.8	4.3	
Chronic nephropathies (%)	11.7	15.0	8.5	6.5	*	5.2	6.9	4.8	3.8	*	18.9	19.0	18.9	18.6	
NSTEMI (%)	53.9	58.9	48.9	46.7	*	47.4	53.8	44.6	43.5	*	61.3	61.4	60.9	60.5	
Previous AMI episodes (%)	9.4	10.0	9.2	7.9	*	8.1	8.8	8.0	7.3	*	10.9	10.6	12.6	10.3	
30-day mortality (%)	7.0	9.8	4.3	2.8	*	2.5	3.5	2.4	1.6	*	12.1	13.0	9.8	8.2	*

*p* < 0.05 in test for trend between levels of education

Low education = no middle school diploma, mid education = middle school diploma, and high education = high school diploma or more

**Table 2:** Readmission analysis: baseline characteristics of study population, stratified by age and education

	Overall					age ≤75					age > 75				
	Level of education					Level of education					Level of education				
	All	Low	Mid	High	*p*	All	Low	Mid	High	*p*	All	Low	Mid	High	*p*
Number	22,181	11,692	6451	4038		12,174	4020	4834	3320		10,007	7672	1617	718	
%	100	52.7	29.1	18.2		100	33.0	39.7	27.3		100	76.7	16.2	7.2	
Demographic variables															
Age in years	71.2	77.6	65.2	62.1	*	61.5	66.5	59.9	57.9	*	82.9	83.4	81.4	81.8	*
Male (%)	64.4	54.1	73.6	79.5	*	76.2	68.3	78.6	82.3	*	50.0	46.6	58.5	67.0	*
Clinical variables															
Tumours (%)	5.3	5.8	5.0	4.5	*	4.3	5.0	4.2	3.6	*	6.6	6.2	7.3	8.9	*
Diabetes (%)	8.4	10.5	7.3	4.2	*	6.0	8.7	5.7	3.2	*	11.3	11.4	12.2	8.6	
Hematologic diseases (%)	6.4	8.7	4.4	3.0	*	2.8	3.8	2.6	2.0	*	10.8	11.2	9.9	7.5	*
Hypertensive diseases (%)	14.0	17.0	11.8	8.8	*	9.3	11.5	9.0	7.0	*	19.7	19.9	19.9	17.1	
Other forms of ischemic heart diseases (%)	11.4	13.6	9.9	7.7	*	8.0	9.9	7.6	6.2	*	15.6	15.5	16.7	14.3	
Previous heart failures (%)	6.3	8.6	4.3	2.9	*	2.9	4.2	2.4	1.9	*	10.5	10.9	9.8	7.4	*
Other cardiac conditions (%)	2.6	3.2	1.9	1.8	*	1.3	2.0	1.2	0.8	*	4.0	3.8	4.3	6.0	*
Conduction disorders and cardiac dysrhythmias (%)	6.3	8.5	4.5	3.0	*	2.8	4.2	2.4	1.7	*	10.6	10.7	11.0	8.8	
Cerebrovascular diseases (%)	8.8	11.2	7.0	4.8	*	4.6	6.1	4.3	3.1	*	14.0	13.9	15.0	12.3	
Vascular diseases (%)	7.1	8.4	6.2	4.7	*	5.6	7.3	5.3	3.9	*	8.9	9.0	9.1	8.1	
Chronic obstructive pulmonary disease (%)	4.0	5.2	3.3	1.7	*	2.3	3.4	2.1	1.2	*	6.1	6.2	6.7	4.3	
Chronic nephropathies (%)	11.1	14.5	8.1	6.2	*	5.1	6.7	4.7	3.7	*	18.5	18.6	18.4	18.1	
NSTEMI (%)	55.0	60.8	49.5	47.1	*	47.7	54.3	45.0	43.8	*	63.8	64.1	62.8	62.0	
Previous AMI episodes (%)	9.4	10.1	9.1	7.9	*	8.0	8.8	8.0	7.3	*	11.1	10.8	12.6	10.4	
30-day readmission (%)	12.3	14.4	10.5	9.2	*	9.6	11.5	8.7	8.6	*	15.7	16.0	15.8	12.0	*

*p* < 0.05 in test for trend between levels of education

Low education = no middle school diploma, mid education = middle school diploma, and high education = high school diploma or more

### Mortality analysis

Educational inequalities were found in 30-day mortality for AMI patients hospitalized in Tuscany. In both unadjusted and full-adjusted models, educational inequalities among patients under 75 years were stronger than those over 75 years.

In the unadjusted model (model 1), both patients with mid and high education had lower odds of 30-day mortality than patients with low education. In particular, patients under 75 years with a high education had a 57% decreased odds of 30-day mortality compared to those under 75 years with a low education. Patients over 75 years with a high education had a 40% decreased odds of 30-day mortality compared to those over 75 years with a low education. After adjusting for demographic and clinical characteristics (model 2), the educational inequalities were attenuated, and remained significant only by comparing the higher educated group with the lower one (OR age ≤ 75 0.68, 95% CI: 0.48–0.96; OR age > 75 0.72, 95% CI: 0.54–0.95). In the full-adjusted model (model 3), there was no relevant change in the association between educational level and 30-day mortality (OR age ≤ 75 0.67, 95% CI: 0.47–0.94; OR age > 75 0.72, 95% CI: 0.54–0.95).

### Readmission analysis

Unlike the mortality analysis, in the readmission analysis we found a significant association between educational level and short-term readmission only among patients aged over 75 years.

**Fig. 2:**
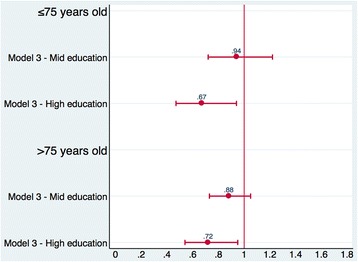
Odds ratios (95% CIs) for 30-day mortality. Legend: Odds ratios and 95% confidence intervals reported from multilevel logistic regressions for 30-day mortality. Low education is the reference category, which is compared with mid education and high education. Model 3 = model adjusted for patients’ age, sex, and clinical characteristics, and for hospital characteristics. Results are reported for patients aged both under and over 75 years

**Fig. 3:**
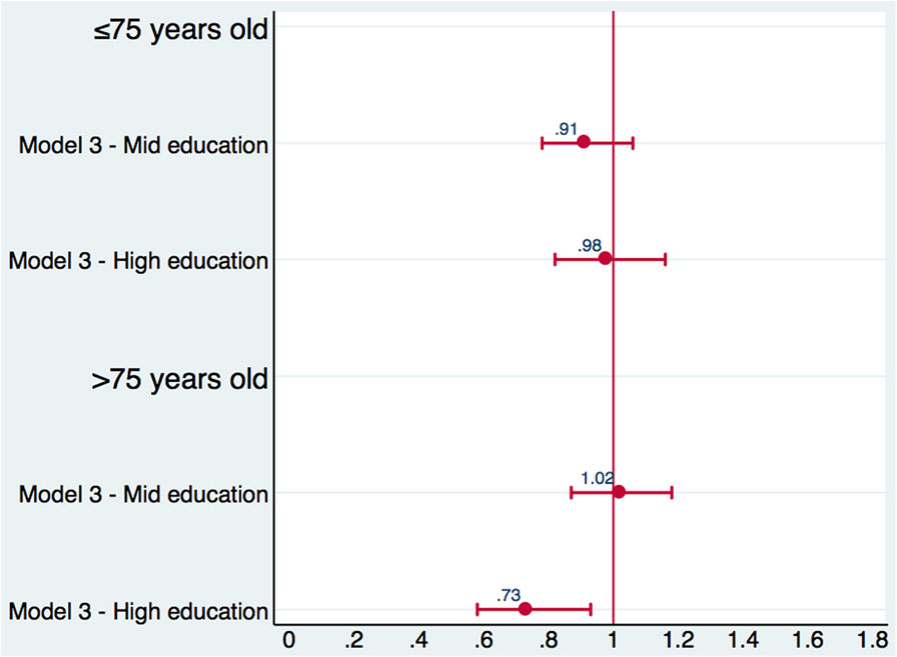
Odds ratios (95% CIs) for 30-day readmission. Legend: Odds ratios and 95% confidence intervals reported from multilevel logistic regressions for 30-day readmission. Low education is the reference category, which is compared with mid education and high education. Model 3 = model adjusted for patients’ age, sex, and clinical characteristics, and for hospital characteristics. Results are reported for patients aged both under and over 75 year

In the unadjusted model (model 1), the association between educational level and short-term readmission was significant for both age groups. Patients hospitalized for AMI aged both under and over 75 years with a high education had a 22 and 28% decreased odds of 30-day readmission, respectively compared to those patients with a low education. After adjusting for patient’s demographic and clinical characteristics (model 2) and hospital characteristics (model 3), the association was not attenuated and remained significant only among the older group. In the full-adjusted model, patients aged over 75 years with a high education had a 27% decreased odds of 30-day mortality compared to those patients aged over 75 years with a low education (OR age > 75 0.73, 95% CI: 0.58–0.93). The results are shown in Table 3 and 4, and Figs. 2 and 3.

**Table 3:** Odds ratios and 95% confidence intervals for 30-day outcomes after hospitalization for AMI

	30-day mortality (*N* = 12,409)			30-day readmission (*N* = 12,174)		
	Model 1	Model 2	Model 3	Model 1	Model 2	Model 3
Low education	1	1	1	1	1	1
Mid education	0.68 (0.53–0.87)	0.94 (0.72–1.22)	0.94 (0.72–1.22)	0.77 (0.66–0.88)	0.91 (0.78–1.06)	0.91 (0.78–1.06)
High education	0.43 (0.31–0.59)	0.68 (0.48–0.96)	0.67 (0.47 0.94)	0.78 (0.66–0.92)	0.98 (0.82–1.16)	0.98 (0.82–1.16)
Male	–	0.91 (0.70–1.19)	0.91 (0.70–1.18)	–	0.92 (0.80–1.06)	0.92 (0.80–1.07)
Age	–	1.05 (1.03–1.07)	1.05 (1.03–1.07)	–	1.02 (1.01–1.03)	1.02 (1.01–1.03)
Tumours	–	4.00 (2.91–5.50)	3.99 (2.91–5.48)	–	1.04 (0.78–1.38)	1.04 (0.78–1.38)
Diabetes	–	1.26 (0.82–1.92)	1.23 (0.81–1.88)	–	1.18 (0.90–1.53)	1.18 (0.90–1.53)
Hematologic diseases	–	2.08 (1.36–3.18)	2.08 (1.36–3.17)	–	1.13 (0.80–1.58)	1.12 (0.80–1.57)
Hypertensive diseases	–	0.87 (0.59–1.28)	0.87 (0.59–1.29)	–	1.07 (0.84–1.36)	1.07 (0.84–1.36)
Other forms of ischemic heart diseases	–	0.85 (0.55–1.32)	0.86 (0.55–1.32)	–	0.96 (0.75–1.24)	0.96 (0.74–1.24)
Previous heart failures	–	1.79 (1.06–3.04)	1.80 (1.06–3.06)	–	1.55 (1.11–2.18)	1.54 (1.10–2.17)
Other cardiac conditions	–	1.07 (0.50–2.28)	1.08 (0.50–2.29)	–	0.96 (0.59–1.55)	0.96 (0.59–1.55)
Conduction disorders and cardiac dysrhythmias	–	1.45 (0.86–2.44)	1.41 (0.84–2.39)	–	0.88 (0.62–1.26)	0.89 (0.62–1.26)
Cerebrovascular diseases	–	1.99 (1.35–2.93)	1.98 (1.35–2.92)	–	0.87 (0.66–1.16)	0.87 (0.65–1.15)
Vascular diseases	–	1.97 (1.38–2.82)	1.95 (1.36–2.78)	–	1.35 (1.06–1.72)	1.35 (1.06–1.72)
Chronic obstructive pulmonary disease	–	0.97 (0.55–1.68)	0.97 (0.56–1.69)	–	1.28 (0.91–1.82)	1.28 (0.90–1.81)
Chronic nephropathies	–	1.57 (1.05–2.35)	1.61 (1.08–2.40)	–	1.59 (1.25–2.02)	1.59 (1.25–2.03)
NSTEMI	–	0.39 (0.30–0.51)	0.41 (0.31–0.53)	–	1.02 (0.89–1.18)	1.00 (0.87–1.16)
Previous AMI episodes	–	1.07 (0.71–1.61)	1.04 (0.69–1.56)	–	0.94 (0.74–1.18)	0.94 (0.74–1.18)
Cathlab	–	–	0.87 (0.60–1.25)	–	–	0.50 (0.33–0.78)
Teaching	–	–	1.54 (1.15–2.06)	–	–	0.88 (0.50–1.57)

Odds ratios reported from multilevel logistic regressions, the reference category is low education

Model 1: unadjusted

Model 2: model 1 adjusted for patients’ age, sex, and clinical characteristics

Model 3: model 2 adjusted for hospital characteristics

Patients ≤ 75 years old

**Table 4:** Odds ratios and 95% confidence intervals for 30-day outcomes after hospitalization for AMI

	30-day mortality (*N* = 10,993)			30-day readmission (*N* = 10,007)		
	Model 1	Model 2	Model 3	Model 1	Model 2	Model 3
Low education	1	1	1	1	1	1
Mid education	0.74 (0.62–0.88)	0.87 (0.73–1.05)	0.88 (0.73–1.05)	1.03 (0.88–1.20)	1.02 (0.87–1.19)	1.02 (0.87–1.18)
High education	0.60 (0.46–0.79)	0.72 (0.54–0.95)	0.72 (0.54–0.95)	0.72 (0.57–0.91)	0.73 (0.57–0.93)	0.73 (0.58–0.93)
Male	–	1.05 (0.93–1.20)	1.06 (0.93–1.20)	–	1.05 (0.94–1.18)	1.05 (0.94–1.18)
Age	–	1.11 (1.10–1.12)	1.11 (1.10–1.12)	–	1.00 (0.99–1.01)	1.00 (0.99–1.01)
Tumours	–	1.86 (1.50–2.29)	1.85 (1.50–2.28)	–	1.09 (0.88–1.35)	1.09 (0.88–1.35)
Diabetes	–	1.35 (1.11–1.64)	1.35 (1.11–1.64)	–	1.05 (0.87–1.26)	1.05 (0.87–1.26)
Hematologic diseases	–	0.98 (0.81–1.18)	0.98 (0.81–1.18)	–	0.92 (0.77–1.11)	0.92 (0.77–1.11)
Hypertensive diseases	–	0.78 (0.66–0.93)	0.78 (0.66–0.93)	–	1.01 (0.86–1.18)	1.01 (0.87–1.18)
Other forms of ischemic heart diseases	–	0.94 (0.78–1.14)	0.94 (0.78–1.14)	–	1.15 (0.97–1.36)	1.15 (0.97–1.36)
Previous heart failures	–	1.78 (1.46–2.16)	1.77 (1.46–2.16)	–	1.22 (1.01–1.47)	1.22 (1.01–1.47)
Other cardiac conditions	–	0.99 (0.72–1.35)	0.98 (0.72–1.34)	–	0.73 (0.54–0.98)	0.72 (0.54–0.98)
Conduction disorders and cardiac dysrhythmias	–	1.39 (1.15–1.68)	1.38 (1.15–1.67)	–	1.17 (0.98–1.40)	1.17 (0.98–1.40)
Cerebrovascular diseases	–	1.56 (1.33–1.83)	1.55 (1.32–1.82)	–	1.16 (0.99–1.35)	1.15 (0.99–1.35)
Vascular diseases	–	1.04 (0.84–1.30)	1.04 (0.84–1.30)	–	1.10 (0.91–1.33)	1.10 (0.91–1.33)
Chronic obstructive pulmonary disease	–	1.19 (0.94–1.50)	1.18 (0.94–1.50)	–	1.03 (0.83–1.29)	1.03 (0.82–1.29)
Chronic nephropathies	–	1.23 (1.05–1.43)	1.23 (1.05–1.43)	–	1.28 (1.11–1.48)	1.28 (1.11–1.47)
NSTEMI	–	0.35 (0.30–0.39)	0.34 (0.30–0.39)	–	0.93 (0.82–1.05)	0.91 (0.81–1.03)
Previous AMI episodes	–	0.83 (0.67–1.03)	0.83 (0.67–1.03)	–	1.03 (0.86–1.23)	1.03 (0.86–1.23)
Cathlab	–	–	0.79 (0.63–0.98)	–	–	0.72 (0.55–0.94)
Teaching	–	–	1.06 (0.81–1.40)	–	–	0.95 (0.66–1.36)

Odds ratios reported from multilevel logistic regressions, the reference category is low education

Model 1: unadjusted

Model 2: model 1 adjusted for patients’ age, sex, and clinical characteristics

Model 3: model 2 adjusted for hospital characteristics

Patients > 75 years old

## Discussion

This study found that patients with a low education are more likely to die during the 30-days following hospitalization for AMI compared to patients with a high education, while only older patients with a low education are more likely to be readmitted in Tuscany. The association between educational level and short-term outcomes remained significant after adjusting for patient and hospital variables.

We decided to stratify our analysis instead of using models that interact age with educational attainment because we were interested in presenting the effect of SES on short-term outcomes for the elderly, thereby covering a gap in the literature. A recent literature review on SES and hospital readmissions for heart failure and AMI [34] highlighted the need for more studies that focus on specific population of elderly patients. Stratification with the cut point at the age 75 allowed us to focus on the very elderly patients, who are more likely to be readmitted due to preventable patient-related response, such as lower self-management capacity after discharge [35–37]. As explained in detail below, differences in patient-related responses after hospitalization for AMI is considered as a possible explanatory mechanism for educational inequalities in short-term outcomes. Other studies on risk factors for hospital readmissions focused on patients aged more than 75 years for similar reasons [38–40].

Assessing the causal path between SES and short-term mortality and readmission was out of the scope of this study and we were unable to disentangle the explanatory mechanisms for educational inequalities in short-term outcomes, due to a lack of data. However, based on the existing related frameworks, we identify three possible explanatory mechanisms.

First, higher short-term mortality and readmission for less-educated AMI patients could be explained by differences in lifestyle, such as smoking, sleeping well, alcohol consumption, and amount of exercise. Patients with a higher SES are more likely to have a healthy lifestyle compared to patients with a lower SES [41, 42].

Second, inequalities in short-term outcomes might be caused by differences in acute care treatment [16]. In Denmark, for example, Rasmussen et al. found that patients with a higher SES were more likely to be performed a coronary artery bypass grafting compared to patients with a lower SES [43].

Third, differences in patient-related responses after hospitalization for AMI are also considered as a possible explanatory mechanism [9, 35, 36, 44–46]. Differences in patient-related responses are associated with lower social capital and social cohesion [44] and lower self-management capacity after discharge [35, 36]. Patients with a low education often have poor self-management skills, probably because they are less aware of the severity of symptoms and therapeutic measures and, consequently, less likely to turn instructions into practical measures, adhere to medication regimens, or obtaining follow-up care [45, 46]. Differences in patient-related responses after hospitalization for AMI appear to be particularly important in explaining readmission. Arbaje et al. found that patients lacking self-management skills were more likely to be readmitted within 60 days after discharge among community-dwelling Medicare beneficiaries [35]. Hence, the fact that older patients have a lower self-management capacity and less social cohesion compared to younger patients [37] could justify our significant association between education and short-term readmission limited to patients over 75 years.

### Comparison with other studies

The association between SES and short-term mortality has been examined using various SES measures, such as education, income [9], occupation [47], and deprivation [14]. We compared our findings above all with studies on education and short-term mortality conducted in European welfare state countries with universal health care systems, like the Italian one. Note that crude 30-day mortality after hospitalization for AMI in Tuscany (7.0%) is lower than the average 30-day mortality in Europe, according to the OECD data (10.8%) [48].

We identified studies conducted in Norway [16], Denmark [17], Finland [18, 19], and Italy [14, 15]. Overall, our findings are consistent with existing evidence, which showed a higher risk of death for lower educated patients, despite the efforts to promote equal access to health care typical of universal health care systems. Interestingly, the only studies showing a weak and inconsistent effect of individual education on short-term fatality were conducted in Italy [14, 15]. However, other Italian studies, which used SES indicators but not education, confirmed SES inequalities in patients with AMI [47, 49, 50].

Similarly to the Finnish study [18], the effect of education on mortality was weaker for the older age group, but remained significant. The attenuation in the effect for the older group might be related to scarce prevention and unhealthy lifestyles at the time when older patients had been attending school. In addition, patients who survived until at least 75 years might be considered healthier than patients who died before, which might have reduced the effect of education on mortality among older patients (survival effect) [16]. In Norway and Denmark, a significant association was found only in the younger group of patients, while in our study it was found in both age groups. This could be explained by differences across the studies in the selection of the cut-off for the division of patients by age and in the definition of categories for the levels of education. We decided to consider both patients with a high school diploma and a university degree as high-educated patients, on the basis of our birth cohort [30].

To the best of our knowledge, no study has been published on education and short-term readmission in Europe. Available evidence is limited to the United States (US) and results are inconsistent. According to a systematic review on the influence of SES on hospital readmissions for heart failure and AMI in patients older than 65 years [34], educational status was not associated with short-term readmission, based on the results of the US studies examined [51, 52]. However, other US studies showed that having a limited education was one of the factors associated with an increased risk of 30-day readmission [4].

### Policy implications

Neither the measurement of inequalities [25] nor the impact of SES variables on quality performance measures have yet gained major managerial attention at the local or regional level. 30-day mortality and readmission are considered as performance measures at the hospital level. Recognizing the role of individual SES contributes to making hospitals aware of the influence of SES on their health care outcomes, which are subject to performance evaluation. This awareness could motivate hospitals to improve their performance outcomes through strategies that take into account individual SES.

Our findings suggest that the educational component should not be underestimated in attempts to improve short-term outcomes after hospitalization for AMI and decrease unnecessary costs [4]. Particular attention should be given to older patients with low education in order to reduce early readmission. Note that, over the study period, the estimated cost of unplanned readmissions to hospitals in Tuscany taken as a whole was €9,196,891. This crude estimation is derived by multiplying the total number of unplanned readmissions of our cohort of AMI patients during the study period by the median readmission cost as measured in terms of diagnosis-related group tariffs.

Hospital managers and policy makers, especially in universal health care systems, might be interested in strategies that are sensitive to low-educated patients in order to improve 30-day outcomes following hospitalization for AMI. These strategies include: promotion of healthier life styles, hospital-initiated transitional care [53, 54], and post-hospitalization support, such as follow-up for disadvantaged patients [55] and patient-centred adherence intervention [56]. These strategies are not mutually exclusive and their effectiveness finds support in the literature. Given the scope of this study and the data at our disposal, it was impossible to define which strategy should be prioritized by hospital managers and policy makers. However, our results clearly indicate the need for increased attention to elderly patients with lower levels of education, especially regarding 30-day readmission. Post-hospitalization support strategies have the potential to be particularly effective for elderly patients with less education, because they increase patient understanding of symptoms and therapeutic measures, and adherence to medication regimens, thus influencing patient response after discharge.

Future research is needed to support hospital managers and policy makers in defining which strategy sensitive to low-educated patients should be prioritized. In this sense, studies on the comparative effectiveness of different strategies would be useful, as well as qualitative studies to disentangle the explanatory mechanisms for educational inequalities in short-term outcomes.

Our analysis also supports Damiani et al. [34] who claimed for the introduction of indicators to measure and understand the role of SES inequalities in the risk for mortality and readmission. In particular, supplementing unadjusted measures with outcomes stratified by SES groups might be useful in motivating quality improvements at the hospital level and targeting efforts towards disadvantaged patients [57].

### Strengths and limitations

This paper has several strengths. First, to the best of our knowledge, this is the first study in Europe exploring the association between individual education and 30-day readmission after hospitalization for AMI, which is particularly important in view of the limited understanding and the increased interest in this hospital quality indicator [58]. Second, we were able to include both individual variables (socio-demographic and clinical) and hospital variables in our models. As a consequence, similar to the most robust studies [8, 9, 59], it was possible to adjust the associations between educational level and short-term outcomes for the hospital characteristics. Third, educational status was measured at the individual level and was divided into three categories, taking into account the birth cohort of AMI patients for the definition of categories. By using an individual-level indicator instead of an area-based measure, we avoided the ecological fallacy. Note that information on education registered at HDRs has been considered as valid and fairly reliable [60].

This study has also some limitations. First, because data were collected from HDRs, it was impossible to consider all potential confounding variables. As with other studies using administrative data [9, 16], we lacked information on AMI severity, lifestyle factors such as smoking or alcohol consumption, treatment adherence, and procedural characteristics. Second, it is possible that comorbidities are unreported in some hospitals, which could lead to an overestimation of the educational effect [27]. Third, although education proved to be a good SES proxy for AMI short-term outcomes, our assessment of SES was based entirely on education [21]. We could not consider SES variables other than education, nor the interactions among them, which could provide a more comprehensive picture of individual levels of SES.

## Conclusions

We found an association between educational level and 30-day mortality and readmission among patients hospitalized for AMI in Tuscany. For the 30-day readmission analysis, a significant association was limited to patients over 75 years. Our results suggest that hospitals might consider strategies that are sensitive to low SES patients to improve both health equity and their performance measures. Further research is needed to provide comparable evidence in other European universal health care systems, especially in terms of readmission analysis, and to assess the association between SES and other outcomes, considered as quality indicators at the hospital level.

## Abbreviations

AMI: Acute myocardial infarction
CI: Confidence interval
HDR: Hospital discharge record
ICD-9-CM: International classification of diseases, 9th revision, clinical modification
OECD: Organization for Economic Co-operation and Development
OR: Odds ratio
PES: Performance evaluation system
SES: Socioeconomic status
US: United States
